# Biphasic Regulation of Caveolin-1 Gene Expression by Fluoxetine in Astrocytes: Opposite Effects of PI3K/AKT and MAPK/ERK Signaling Pathways on c-fos

**DOI:** 10.3389/fncel.2017.00335

**Published:** 2017-10-31

**Authors:** Baoman Li, Shu Jia, Tingting Yue, Li Yang, Chen Huang, Alexej Verkhratsky, Liang Peng

**Affiliations:** ^1^Laboratory of Metabolic Brain Diseases, Institute of Metabolic Disease Research and Drug Development, China Medical University, Shenyang, China; ^2^Faculty of Life Science, The University of Manchester, Manchester, United Kingdom; ^3^Achucarro Center for Neuroscience, IKERBASQUE, Basque Foundation for Science, Bilbao, Spain

**Keywords:** astrocyte, Cav-1, cfos, PI3K/AKT, MAPK/ERK

## Abstract

Previously, we reported that fluoxetine acts on 5-HT_2B_ receptor and induces epidermal growth factor receptor (EGFR) transactivation in astrocytes. Recently, we have found that chronic treatment with fluoxetine regulates Caveolin-1 (Cav-1)/PTEN/PI3K/AKT/glycogen synthase kinase 3β (GSK-3β) signaling pathway and glycogen content in primary cultures of astrocytes with bi-phasic concentration dependence. At low concentrations fluoxetine down-regulates Cav-1 gene expression, decreases membrane content of PTEN, increases PI3K activity and increases phosphorylation of GSK-3β and increases its activity; at high concentrations fluoxetine acts on PTEN/PI3K/AKT/GSK-3β in an inverse fashion. Here, we present the data indicating that acute treatment with fluoxetine at lower concentrations down-regulates c-Fos gene expression via PI3K/AKT signaling pathway; in contrast at higher concentrations fluoxetine up-regulates c-Fos gene expression via MAPK/extracellular-regulated kinase (ERK) signaling pathway. However, acute treatment with fluoxetine has no effect on Cav-1 protein content. Similarly, chronic effects of fluoxetine on Cav-1 gene expression are suppressed by inhibitor of PI3K at lower concentrations, but by inhibitor of MAPK at higher concentrations, indicating that the mechanism underlying bi-phasic regulation of Cav-1 gene expression by fluoxetine is opposing effects of PI3K/AKT and MAPK/ERK signal pathways on c-Fos gene expression. The effects of fluoxetine on Cav-1 gene expression at both lower and higher concentrations are abolished by AG1478, an inhibitor of EGFR, indicating the involvement of 5-HT_2B_ receptor induced EGFR transactivation as we reported previously. However, PP1, an inhibitor of Src only abolished the effect by lower concentrations, suggesting the relevance of Src with PI3K/AKT signal pathway during activation of EGFR.

## Introduction

Astroglial contribution to major depression (similarly to other psychiatric pathologies—see Verkhratsky et al., 2014) does not involve astrogliosis and hypertrophy being mainly manifested by a decrease in astroglial numbers and their hypotrophy. Previously we found that fluoxetine, as well as other selective serotonin reuptake inhibitors (SSRIs) act as agonists of 5-HT_2B_ receptors in astrocytes (Li et al., 2008; Zhang et al., 2010; Peng et al., 2016). Astrocytes in cultures and in the brain *in vivo* express high level of 5-HT_2B_ receptors (Peng and Huang, 2012). The 5-HT_2B_ receptors are Gq/11 protein coupled and stimulation of these receptors generates diacyglycerol (DAG) and inositol 1,4,5-triphosphate (InsP_3_). The latter triggers an increase of intracellular calcium concentration ([Ca^2+^]_i_), which in turn activates Zn^2+^-dependent metalloproteinases and leads to shedding of growth factor(s) (for review see Peng and Huang, 2012). Subsequently, the released growth factors activate epidermal growth factor receptors (EGFRs). The downstream target of EGFR, extracellular-regulated kinase (ERK) is phosphorylated via the Ras/Raf/MEK pathway, and AKT is phosphorylated via PI3K pathway. Phosphorylation of AKT and ERK occurs in a few minutes after fluoxetine administration, and lasts only for 40 min (Li et al., 2008; Bai et al., 2017). However, both pathways may induce long-term modification of astrocytic functions via regulation of gene expression (Li et al., 2008; Hertz et al., 2012, 2014).

Caveolin-1 (Cav-1), a scaffolding/regulatory protein, is an essential structural constituent of caveolae, which are flask-shaped invaginations of cell membrane (Lajoie and Nabi, 2010; Takizawa et al., 2013). The putative functions of Cav-1 are cholesterol transport (Yue and Mazzone, 2011) and endocytosis (Moskovich et al., 2012). In addition, Cav-1 modulates signal transduction by linking signaling molecules and thus regulating their downstream activity (Zebrowski et al., 1994). Caveolae, as well as caveolin protein are present in astrocytes (Cameron et al., 1997) where they contribute to lipid metabolism, endocytosis and signal transduction (Silva et al., 2007). Recently, we have found that chronic treatment with fluoxetine modifies Cav-1/PTEN/PI3K/AKT/GSK-3β signaling pathway (where PTEN stands for phosphatase and tensin homolog, and GSK-3 for glycogen synthase kinase 3) in primary cultures of astrocytes with bi-phasic concentration dependence (Bai et al., 2017). The GSK-3, the downstream substrate of AKT, is an enzyme, which has been initially discovered as a deactivator of glycogen synthase (GS) that converts glucose to glycogen. In addition, GSK-3β is involved in diverse signaling pathways. At lower concentrations fluoxetine down-regulates gene expression of Cav-1. The Cav-1 contains sequences for PTEN binding (Xia et al., 2010), and hence the down-regulation of Cav-1 expression reduces membrane content of PTEN, increases activity of PI3K/AKT, and elevates GSK-3β phosphorylation thus suppressing its activity. At higher concentrations fluoxetine acted in an inverse fashion (Bai et al., 2017). This outcome of chronic treatment is distinct from the acute effects of fluoxetine on AKT and ERK phosphorylation, which is directly proportional to the concentration of the drug (Li et al., 2008; Bai et al., 2017).

To understand mechanisms underlying bi-phasic concentration dependence of Cav-1 expression by fluoxetine, we have investigated: (i) effects of inhibitors of PI3K and MAPK on AKT and ERK phosphorylation induced by acute treatment with fluoxetine; (ii) effects of inhibitors of PI3K and MAPK on mRNA and protein expression of cFos and FosB in response to acute treatment with fluoxetine; (iii) effects of inhibitors of EGFR and Src on expression of Cav-1 mRNA and protein during chronic treatment with fluoxtine; (iv) effects of inhibitors of PI3K and MAPK on mRNA and protein expression of Cav-1 mRNA and protein in response to chronic treatment with fluoxtine; and (v) effects of inhibitors of PI3K and MAPK on glycogen content in response to chronic treatment with fluoxtine in primary astroglial cultures.

## Materials and Methods

### Animals

Newborn CD-1 mice (Charles River, Beijing, China) were used for primary cultures of astrocytes. All experiments were carried out in accordance with the USA National Institute of Health Guide for the Care and Use of Laboratory Animals, and all experimental protocols were approved by the Institutional Animal Care and Use Committee of China Medical University.

### Cell Cultures

Primary cultures of astrocytes were prepared as previously described (Hertz et al., 1998). The neopallia of the cerebral hemispheres of newborn CD-1 mice were aseptically isolated, vortexed to dissociate the tissue, filtered through nylon meshes with pore sizes of 80 μm and subsequently 10 μm, diluted in culture medium, and planted in Falcon Primaria culture dishes. The culture medium was a Dulbecco’s Minimum Essential Medium (DMEM) with 7.5 mM glucose, initially containing 20% horse serum, and the cultures were incubated at 37°C in a humidified atmosphere of CO_2_/air (5:95%). The culture medium was exchanged with fresh medium of similar composition on day 3, and subsequently every 3–4 days. From day 3, the serum concentration was reduced to 10%, and after the age of 2 weeks, 0.25 mM dibutyryl cyclic AMP (dBcAMP) was included in the medium, leading to morphological and functional differentiation (Meier et al., 1991). The cultures were used after at least 3 weeks of culture.

### Drug Treatment

#### Acute Drug Treatment

For determination of phosphorylation of ERK_1/2_ and AKT, gene expression of c-fos and fosB, and Cav-1 protein content, cells were incubated in the culturing medium without serum at 37°C for 20 min, 1 h or 4 h in the absence or presence of 0.1, 0.5, 1, 5, or 10 μM fluoxetine.

#### Chronic Drug Treatment

For determination of gene expression of Cav-1 and glycogen content, cells were treated with fluoxetine at 0, 0.1, 0.5, 1, 5, or 10 μM in the culturing medium with 10% serum at 37°C for 2 weeks.

### Western Blotting

Samples containing 50 μg protein were applied on slab gels of 10% polyacrylamide. The nitrocellulose membranes were incubated with the first antibody, specific to either Cav-1 or β-actin, and specific binding was detected by goat-anti-mouse or goat-anti-rabbit horseradish peroxidase-conjugated secondary antibody (Li et al., 2008). Band density was measured with Window AlphaEaseTM FC 32-bit software. Ratio was determined between scanned Cav-1 and β-actin. Representative original images for the antibodies used in this study are shown in Supplementary Figure S1.

### Reverse Transcription-Polymerase Chain Reaction (RT-PCR)

For determination of mRNA expression by RT-PCR, a cell suspension was prepared, the RNA pellet was precipitated, and RT was performed as previously described (Kong et al., 2002). PCR amplification was performed as described by Li et al. (2008) in a Robocycler thermocycler with sense (5′-ACCTAGCCGTGGAGCTTGG-3′) and antisense (5′-GCCCTTGGTTGTTTACCTGG-3′) for cfos (Elkeles et al., 1999), with sense (5′-AGCTGACAGCATGAAGGTCCTCC-3′) and antisense (5′-TTCTGGGTGAAGACAGAAGGGCC-3′) for fosB (Inoue et al., 2004), with sense (5′-CTACAAGCCCAACAACAAGGC-3′) and antisense (5′-AGGAAGCTCTTGATGCACGGT-3′) for Cav-1 (Hsieh et al., 2013) or with sense (5′-CCACGGACAACTGCGTTGAT-3′) and antisense (5′-GGCTCATAGCTACTGAACTG-3′) for TBP (Marjou et al., 2000), used as a housekeeping gene.

### Determination of Glycogen

For determination of glycogen in cultured astrocytes, recording fluorescence intensity of NADPH generated from NADP^+^ as our previously described (Bai et al., 2017). Briefly, after 2 weeks treatment with normal saline (NS) or fluoxetine in the absence or presence of U0126 or LY294002, the astrocytes were washed three times with ice-cold phosphate-buffered saline (PBS) and sonicated in 30 mM HCl. The suspension was used to measure non-hydrolyzed glycosyl units of glycogen. The fluorescence of the NADPH formed in amounts equivalent to glucose metabolized by hexokinase was read (excitation 340 nm; emission 450 nm). The sum of glucose and glucose-6-phosphate and the left glycosyl units from glycogen were separately measured, and the difference between these two aliquots was calculated. Meanwhile, the standard curve was made to show fluorescence intensity at different glucose concentrations and glycogen content was calculated which was normalized by protein content (per mg).

### Materials

Chemicals for preparation of medium and most other chemicals, including fluoxetine, first antibody raised against β-actin, LY294002 (2-(4-Morpholinyl)-8-phenyl-1(4H)-benzopyran-4-one hydrochloride), and PP1 (4-amino-5-(4-methylphenyl)-7-(t-butyl)pyrazolo-d-3,4-pyrimidine) were purchased from Sigma (St. Louis, MO, USA). AG 1478 (N-[(2R)-2-(hydroxamidocarbonymethyl)-4-methylpentanoyl]-Ltryptophan methylamide) was obtained from Calbiochem, La Jolla, CA, USA. First antibody raised against Cav-1 was from Cell Signaling Technology (Danvers, MA, USA). HRP conjugated IgG second antibodies and U0126 (1,4-diamino-2,3-dicyano-1,4-bis[2-aminophe-nylthio]butadiene) were from Promega (Madison, WI, USA). ECL detection reagents were from Amersham Biosciences (Buckinghamshire, UK).

### Data Analysis

All values were expressed as mean ± SEM. Data were primarily analyzed for homogeneity of variance using SPSS 17.0 software (Chicago, IL, USA), and *p* values of analysis were >0.05, from 0.373 to 0.722. Statistical significance between groups was determined by a one-way analysis of variance (ANOVA) followed by Fisher’s least significant difference (LSD) for multiple comparisons using GraphPad Prism 5 software (La Jolla, CA, USA). *P* values of less than 0.05 were considered statistically significant.

## Results

### Acute Treatment

#### Phosphorylation of AKT and ERK_1/2_

**Figure 1:**
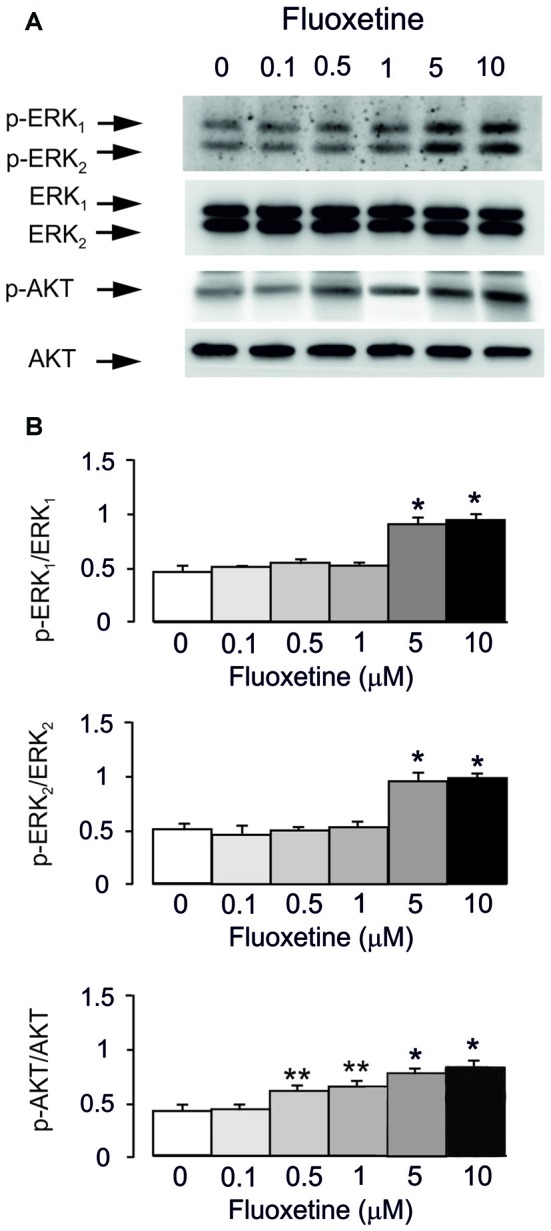
Acute treatment with fluoxetine induced extracellular-regulated kinase (ERK)_1/2_ and AKT phosphorylation in astrocytes. Cells were incubated for 20 min in the absence of any drug (0 μM fluoxetine, control) or in the presence of 0.1, 0.5, 1, 5, or 10 μM fluoxetine. **(A)** Immunoblot from a representative experiment. Similar results were obtained from three independent experiments. Average ERK phosphorylation was quantitated as ratios between p-ERK_1_ and ERK_1_ and between p-ERK_2_ and ERK_2_, or average AKT phosphorylation was quantitated as ratio between p-AKT and AKT **(B)**. SEM values are indicated by vertical bars. *Indicates statistically significant (*P* < 0.05) difference from 0, 0.1, 0.5 and 1 μM fluoxetine; **indicates statistically significant (*P* < 0.05) difference from all other groups but not from each other.

Acute treatment with fluoxetine for 20 min induced phosphorylation of AKT (148% ± 5.2% of control at 0.5 μM and 198% ± 7.5% of control at 10 μM) and ERK_1/2_ (from 196% ± 6.1% of control for ERK_1_ and 184% ± 5.5% for ERK_2_ at 5 μM; from 202% ± 8.3% of control for ERK_1_ and 190% ± 6.9% for ERK_2_ at 10 μM) in a concentration-dependent manner with no effect at 0.1 μM (Figures 1A,B). However, the lowest concentration of fluoxetine stimulating AKT phosphorylation at 0.5 μM is 10 times lower than the concentration significantly stimulating ERK_1/2_ phosphorylation (5 μM). This is in agreement with our previous findings (Li et al., 2008, 2009).

#### mRNA and Protein Expression of c-Fos and FosB

**Figure 2:**
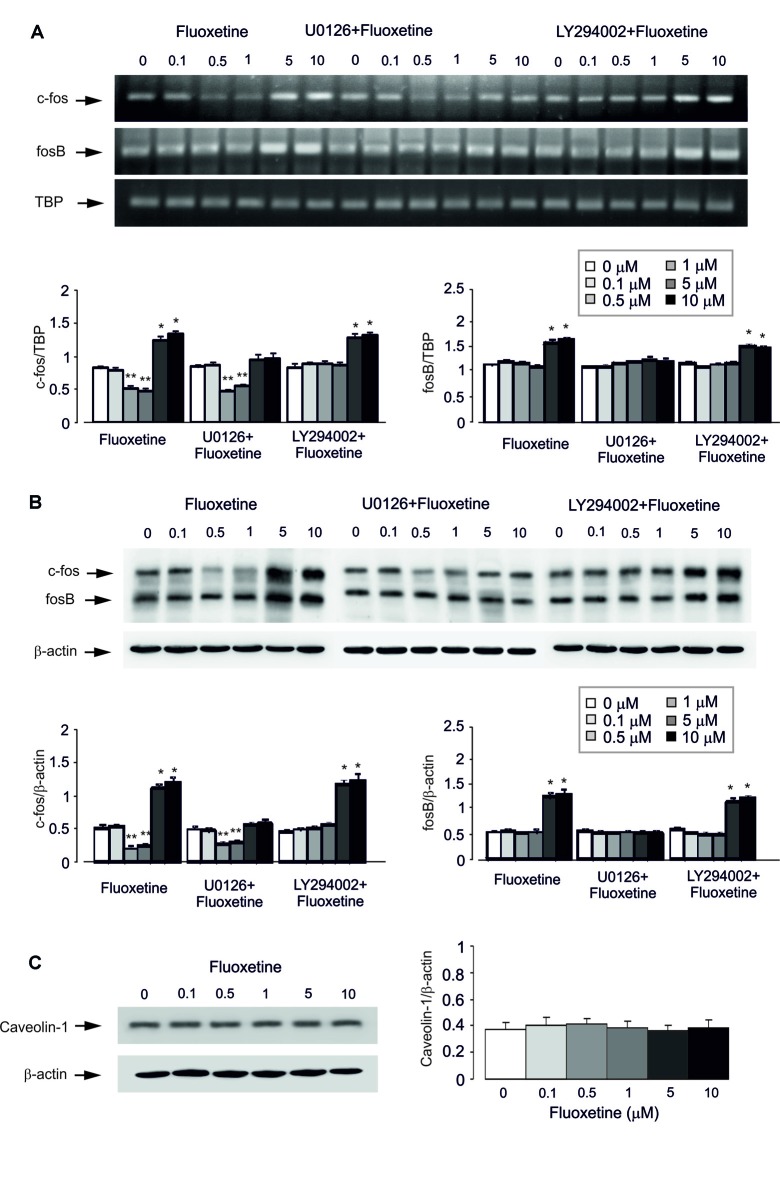
Regulation of mRNA and protein expression of cfos and fosB or protein content of Caveolin-1 (Cav-1) by acute treatment with fluoxetine in astrocytes. **(A,B)** After pretreatment in serum-free medium with or without U0126, an inhibitor of MEK or LY294002, an inhibitor of PI3K for 15 min, cells were incubated for 1 or 4 h in the absence of any drug (0 μM fluoxetine, control) or in the presence of 0.1, 0.5, 1, 5, or 10 μM fluoxetine. **(A)** Southern blot from representative experiment. Similar results were obtained from three independent experiments. Average mRNA expression was quantitated as ratios between c-fos and TBP, used as a house-keeping gene and between fosB and TBP. **(B)** Immunoblot from a representative experiment. Similar results were obtained from three independent experiments. Average protein expression was quantitated as ratios between c-Fos and β-actin, used as a house-keeping gene and between FosB and β-actin. SEM values are indicated by vertical bars. *Indicates statistically significant (*P* < 0.05) difference from 0, 0.1, 0.5 and 1 μM fluoxetine; **indicates statistically significant (*P* < 0.05) difference from all other groups but not from each other. **(C)** Cells were incubated for 1 or 4 h in the absence of any drug (0 μM fluoxetine, control) or in the presence of 0.1, 0.5, 1, 5, or 10 μM fluoxetine. Left panel shows the immunoblot from a representative experiment. Similar results were obtained from three independent experiments. Average protein expression was quantified as ratio between Cav-1 and β-actin, used as a house-keeping gene (right panel). SEM values are indicated by vertical bars.

Acute treatment with fluoxetine for 1 h regulated expression of mRNA for immediate early genes c-Fos in a biphasic concentration-dependent manner, with no effect at 0.1 μM (Figure 2A). Fluoxetine caused significant down-regulation of mRNA for c-Fos at 0.5 and 1 μM whereas at 5 and 10 μM a significant up-regulation of mRNA was observed (Figure 2A). The lowest level of c-Fos expression was detected at 1 μM being 52.6% ± 3.2% of control, whereas the highest level of expression was determined at 10 μM being 147.8% of control group. Expression of mRNA for FosB was up-regulated by fluoxetine at 5 and 10 μM (mRNA levels were increased by 49.4% ± 7.9% and 61.7% ± 5.7% of the control) with no down-regulation at 0.5 and 1 μM. Inhibitor of MAPK U0126 suppressed an increase of mRNA expression of c-Fos and FosB by 5 and 10 μM fluoxetine, but had no effect on the c-Fos down-regulation at 0.5 and 1 μM fluoxetine (Figure 2A). Inhibitor of PI3K LY294002, suppressed the decrease of mRNA expression of c-Fos at 0.5 and 1 μM fluoxetine but had no effect on the increase of mRNA levels at 5 and 10 μM fluoxetine (Figure 2A).

Similar results were also obtained for protein expression of c-Fos and FosB after 4 h treatment with fluoxetine (Figure 2B). Fluoxetine at 0.5 and 1 μM decreased the protein expression of c-Fos, with the lowest expression at 0.5 μM being 40.4% ± 4.7% of control group, but at 5 and 10 μM significantly increased the protein level of c-Fos. The highest level of expression was detected at 10 μM being 232.7% ± 9.2% of control group. Again, at lower concentrations, fluoxetine had no inhibitory effect on FosB protein expression. Treatment with U0126 abolished increased protein expression of c-Fos and FosB by 5 and 10 μM fluoxetine, but had no effect on the expression decrease induced by 0.5 and 1 μM fluoxetine. In contrast, LY294002, an inhibitor of PI3K, suppressed the decrease of mRNA expression of c-Fos by 0.5 and 1 μM fluoxetine but had no effect on increased mRNA expression in the presence of 5 and 10 μM fluoxetine (Figure 2B).

#### Protein Content of Cav-1

Acute treatment with fluoxetine at concentrations of 0.1, 0.5, 1, 5, or 10 μM for 1 h had no effect on protein level of Cav-1 (0.1 μM, 113.5% ± 16.2% of control; 0.5 μM, 121.6% ± 10.8% of control; 1 μM, 111% ± 13.5% of control; 5 μM, 94.6% ± 10.7% of control; 10 μM, 105.4% ± 16.2% of control; Figure 2C).

### Chronic Treatment

#### Gene Expression of Cav-1

**Figure 3:**
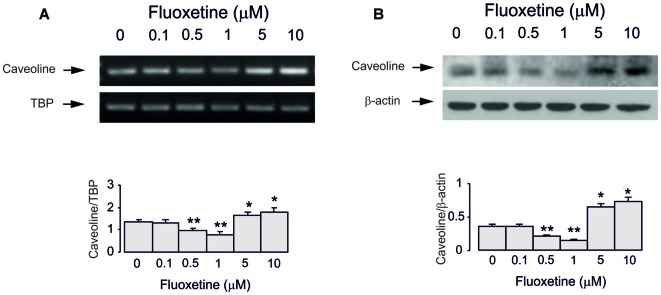
Regulation of mRNA and protein expression of Cav-1 by chronic treatment with fluoxetine in astrocytes. Cells were incubated for 2 weeks in the absence of any drug (0 μM fluoxetine, control) or in the presence of 0.1, 0.5, 1, 5, or 10 μM fluoxetine. **(A)** Southern blot from representative experiment. Similar results were obtained from three independent experiments. Average mRNA expression was quantified as ratio between Cav-1 and TBP, used as a house-keeping gene. **(B)** Immunoblot from a representative experiment. Similar results were obtained from three independent experiments. Average protein expression was quantitated as ratio between Cav-1 and β-actin, used as a house-keeping gene. SEM values are indicated by vertical bars. *Indicates statistically significant (*P* < 0.05) difference from 0, 0.1, 0.5 and 1 μM fluoxetine; **indicates statistically significant (*P* < 0.05) difference from all other groups but not from each other.

Treatment of cultured astrocytes with fluoxetine for 2 weeks regulated expression of Cav-1 protein in a concentration-dependent manner with 0.1, 0.5 and 1 μM fluoxetine causing a significant down-regulation but 5 and 10 μM a significant up-regulation (Figure 3), which agrees well with our previous findings (Bai et al., 2017). The lowest level was observed at 1 μM being 40.9% ± 4.5% of control, whereas the highest level of expression was determined at 10 μM being 137.9% ± 5.7% of control group.

**Figure 4:**
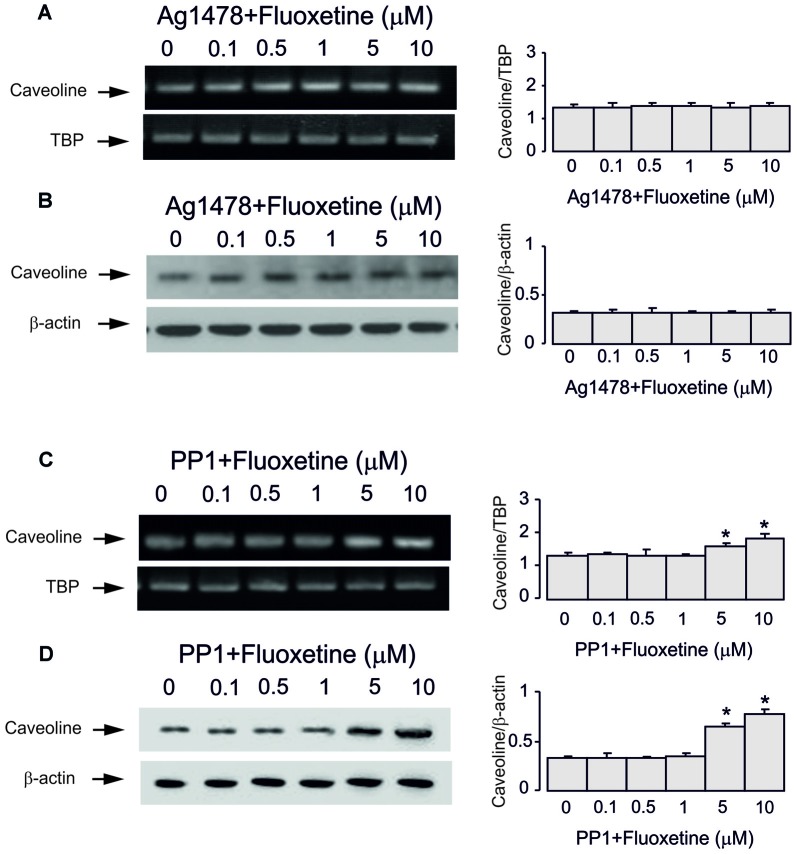
Regulation of mRNA and protein expression of Cav-1 by chronic treatment with fluoxetine requires epidermal growth factor receptor (EGFR) or Src activation in astrocytes. Cells were incubated for 2 weeks without any drug (0 μM fluoxetine, control) or with 0.1, 0.5, 1, 5, or 10 μM fluoxetine in the presence of AG1478, an inhibitor of EGFR or PP1, an inhibitor of Src. **(A,C)** Southern blots from representative experiments. Similar results were obtained from three independent experiments. Average mRNA expression was quantitated as ratio between Cav-1 and TBP, used as a house-keeping gene. **(B,D)** Immunoblots from a representative experiments. Similar results were obtained from three independent experiments. Average protein expression was quantified as ratio between Cav-1 and β-actin, used as a house-keeping gene. SEM values are indicated by vertical bars. *Indicates statistically significant (*P* < 0.05) difference from all other groups but not from each other.

Inhibitor of EGFR AG1478 at 10 μM abolished fluoxetine effects on Cav-1 mRNA and protein expression at both low and high concentrations (0.5 and 1 μM, 101.5% ± 3.1% and 102.2% ± 2.7% of control; 5 and 10 μM, 100.7% ± 4.3% and 101.5% ± 2.9% of control; Figures 4A–D). Inhibitor of Src PP1, at 10 μM suppressed only effects of low concentrations of fluoxetine (0.5 and 1 μM, 100.4% ± 7.2% and 99.6% ± 2.1% of control) without affecting action of high concentrations (5 and 10 μM, 120.7% ± 3.5% and 135.6% ± 5.2% of control).

**Figure 5:**
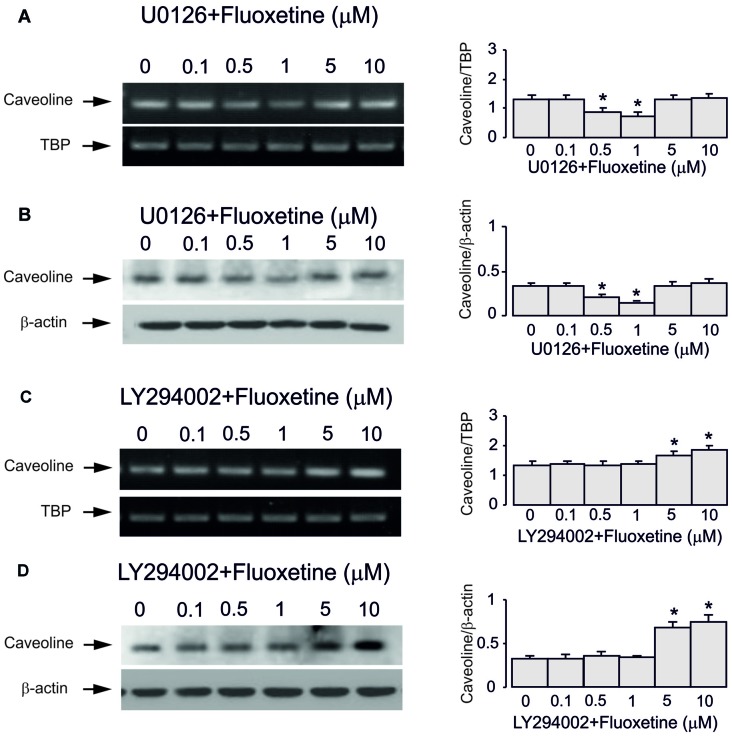
Regulation of mRNA and protein expression of Cav-1 by chronic treatment with fluoxetine requires ERK or AKT activation in astrocytes. Cells were incubated for 2 weeks without any drug (0 μM fluoxetine, control) or with 0.1, 0.5, 1, 5, or 10 μM fluoxetine in the presence of U0126, an inhibitor of MEK or LY294002, an inhibitor of PI3K. **(A,C)** Southern blots from representative experiments. Similar results were obtained from three independent experiments. Average mRNA expression was quantified as ratio between Cav-1 and TBP, used as a house-keeping gene. **(B,D)** Immunoblots from a representative experiments. Similar results were obtained from three independent experiments. Average protein expression was quantified as ratio between Cav-1 and β-actin, used as a house-keeping gene. SEM values are indicated by vertical bars. *Indicates statistically significant (*P* < 0.05) difference from all other groups but not from each other.

Inhibitor of MAPK U0126 at 10 μM did not change fluoxetine effects on Cav-1 mRNA and protein expression at low concentrations (0.5 and 1 μM, 65.7% ± 4.1% and 60.0% ± 4.7% of control) but abolished drug effects at high concentration (5 and 10 μM, 99.3% ± 3.9% and 101.5% ± 3.7% of control; Figures 5A–D). In contrast, an inhibitor of PI3K LY294002 at 10 μM abolished drug effect at low concentrations only (0.5 and 1 μM, 99.3% ± 3.4% and 102.2% ± 2.5% of control) but had no effect on high concentrations (5 and 10 μM, 100.7% ± 3.8% and 101.5% ± 3.5% of control).

#### Glycogen Content

**Figure 6:**
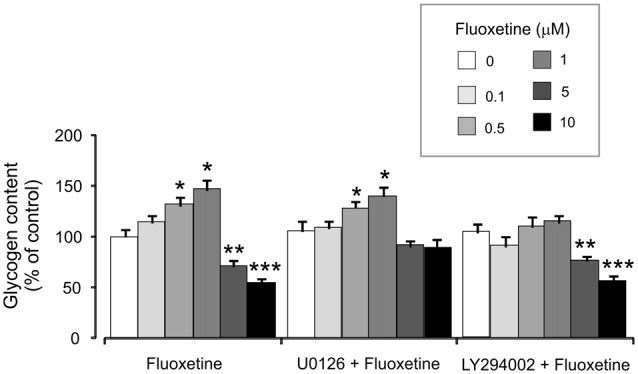
Effect of fluoxetine on the glycogen content requires ERK or AKT activation in astrocytes. Cells were incubated for 2 weeks without any drug (0 μM fluoxetine, control) or with 0.1, 0.5, 1, 5, or 10 μM fluoxetine in the presence of U0126, an inhibitor of MEK or LY294002, an inhibitor of PI3K. After the experiment glycogen was determined by measuring glucose content fluorometrically before and after breakdown of remaining glycogen in astrocytes. Average glycogen contents are indicated as percentages of those under control conditions. SEM values are indicated by vertical bars. *Indicates statistically significant (*P* < 0.05) difference from 0 and 0.1 μM groups but not from each other; **indicates statistically significant (*P* < 0.05) difference from 0, 0.1, 0.5 and 1 μM groups; ***indicates statistically significant (*P* < 0.05) difference from all other groups.

Treatment with 0.5 and 1 μM fluoxetine for 2 weeks increased glycogen content to 132.2% ± 5.1% and 147.7% ± 7.2% of the control group. In contrast, treatment with 5 and 10 μM fluoxetine decreased glycogen content to 72.4% ± 5.3% and 55.2% ± 5.1% of the control (Figure 6). It is in agreement with our previous findings (Bai et al., 2017). Inhibitor of MAPK U0126 at 10 μM did not change fluoxetine effects on glycogen content at low concentrations (0.5 and 1 μM, 130.2% ± 6.2% and 141.2% ± 8.2% of control) but inhibited drug effects at high concentration (5 and 10 μM, 92.3% ± 5.2% and 89.3% ± 9.2% of control). In contrast, an inhibitor of PI3K LY294002 at 10 μM abolished drug effect at low concentrations only (0.5 and 1 μM, 111.4% ± 5.4% and 115.2% ± 7.1% of control) but had no effect on high concentrations (5 and 10 μM, 77.2% ± 4.8% and 57.1% ± 4.1% of control).

## Discussion

Caveolae and caveolin proteins are known to contribute to a various diseases, such as Alzheimer’s disease, Parkinson’s disease, cardiovascular and prion diseases, systemic lupus erythematosus and HIV (Michel et al., 2007). Although no report exists about caveolin contribution to major depression the Cav-1 is linked to schizophrenia in both human polymorphism study (Najafipour et al., 2014) and in Cav-1 knockout animals (Allen et al., 2011). Caveolin proteins are key modulators of a variety of neuronal intracellular signaling pathways (Stern and Mermelstein, 2010). In the brain, Cav-1 is found to play a key role in estrogen-induced, glutamate-independent activation of metabotropic glutamate receptors (mGluRs) that regulate multiple neuronal and glial functions (Meitzen and Mermelstein, 2011). In addition, Cav-1 in astrocytes is associated with reactivity (Niesman et al., 2014), with SorLA, a protein with sorting and trafficking functions and demonstrated relevance to Alzheimer’s disease (Salgado et al., 2012), with expression of connexin Cx43 (Strale et al., 2012), and with fatty acid-binding proteins (FABPs)-related signals (Kagawa et al., 2015). We have reported that chronic treatment of astrocytes with ammonium increases Cav-1 gene expression and membrane PTEN content decreases activity of PI3K/AKT, and suppresses GSK3β phosphrylation that results in the increase of its activity (Wang et al., 2017). Recently, we have found that chronic treatment with fluoxetine regulates Cav-1/PTEN/PI3K/AKT/GSK-3β signaling pathway in primary cultures of astrocytes with bi-phasic concentration dependence (Bai et al., 2017). The GSK-3β is involved in diverse signaling pathways, such as insulin/insulin-like growth factor (IGF-1) signaling, neurotrophic factor signaling, and the Wnt signaling (Gould and Manji, 2005). Since high levels of GSK-3β activity are generally associated with mood disorders (Gould and Manji, 2005), the underlying mechanisms of the bi-phasic concentration dependence become important. In addition, these mechanisms may be also applicable to the regulation of expression of other genes by fluoxetine.

In the present study we found that acute treatment with fluoxetine stimulated AKT phosphorylation at concentrations of 0.5 μM or higher; for ERK_1/2_ phosphorylation the concentration has to be as high as 5 μM, which is in agreement with our previously report (Li et al., 2008, 2009). Chronic treatment of cultured astrocytes with low (0.5–1 μM) concentrations of fluoxetine decreased, whereas chronic treatment with high (5 and 10 μM) concentrations increased expression of Cav-1 mRNA and protein. Inhibitor of EGFR AG1478 abolished drug effects at all concentrations, indicating that both down- and up-regulation of Cav-1 gene expression are mediated by fluoxetine-induced EGFR transactivation, as we reported previously (Li et al., 2008, 2009). In contrast, the Src inhibitor PP1 suppressed fluoxetine effects only at low concentrations, suggesting that at lower concentrations fluoxetine-induced transactivation of EGFR involves Src phosphorylation of Y845, which leads to downstream signaling to the PI3K/AKT pathway (Nair and Sealfon, 2003).

Subsequently, we found that down-regulation of Cav-1 gene expression by lower concentrations of fluoxetine was inhibited by the PI3K inhibitor, LY294002 but not by MAPK inhibitor, U0126; the up-regulation of Cav-1 by higher drug concentrations was inhibited by MAPK inhibitor, U0126 but not by PI3K inhibitor, LY294002. Furthermore, the mRNA expression of c-Fos but not FosB after 1 h treatment and protein expression of c-Fos but not FosB after 4 h treatment were down-regulated by lower concentrations of fluoxetine was inhibited by the PI3K inhibitor, LY294002 but not by MAPK inhibitor, U0126. The up-regulation by higher drug concentrations was suppressed by MAPK inhibitor, U0126 but not by PI3K inhibitor, LY294002. These data suggest that at lower concentrations of fluoxetine AKT inhibits c-Fos gene expression, which in turn, down-regulates Cav-1 gene expression. At higher concentrations ERK_1/2_ stimulates cFos gene expression, which in turn, up-regulates Cav-1 gene expression. Since fluoxetine at lower concentrations has no effect on FosB expression, FosB may not be involved in regulation of Cav-1 gene expression in astrocytes. The distinct effects of AKT and ERK_1/2_ on c-Fos expression was also reported by in human pre-B cells (Anbazhagan et al., 2013). Although it is well known that ERK_1/2_ stimulates c-Fos gene expression, the reports about AKT-induced inhibition are rare and the underlying mechanism is unknown. It has been reported that Cav-1 inhibits Ras/MAPK/ERK_1/2_ cascade (Engelman et al., 1998; Galbiati et al., 1998) and AP-1 transcription factor activation (Engelman et al., 1997; Williams et al., 2004). Further study confirms that Cav-1 reduces activation of ERK_1/2_ that, in turn, decreases expression and activation of c-Fos and c-Jun proteins in PAM212 cells (Trimmer et al., 2013). In the present study, acute treatment with fluoxetine for 1 h had no effect on protein level of Cav-1, suggesting that Cav-1-induced variation of c-fos expression is not involved in the effects of fluoxetine in astrocytes.

The regulation of GSK-3β phosphorylation and activity will change the activity of glycogen synthetase, which, in turn, affects glycogen content in astrocytes. Previously, we found that fluoxetine increased glycogen content in cultured astrocytes at lower concentrations, but decreased it at higher concentrations (Bai et al., 2017). Here, we found that increased glycogen content by lower concentrations of fluoxetine was inhibited by the PI3K inhibitor, LY294002 but not by MAPK inhibitor, U0126; decreased glycogen content by higher drug concentrations was inhibited by MAPK inhibitor, U0126 but not by PI3K inhibitor, LY294002. It is in agreement with the drug effects on Cav-1 gene expression, and further suggests that biphasic regulation of GSK-3β activity by chronic treatment with fluoxetine is via concentration-dependent drug effects on two distinct signal pathways, PI3K/AKT and MAPK/ERK. Regulation of glycogen may contribute to behavioral effects of SSRIs. Glycogen turnover, i.e., interspersed glycogen synthesis and glycogenolysis, is indispensable to support learning (Gibbs and Hutchinson, 2012; Hertz et al., 2013). The acute memory-enhancing, glycogenolysis-dependent effect of both fluoxetine and paroxetine has been characterized (O’Dowd et al., 1994, 1995; Suzuki et al., 2011; Gibbs and Hertz, 2014; Gao et al., 2016). Knock-out of brain GS abolishes learning of new motor and cognitive skills (Duran et al., 2013).

**Figure 7:**
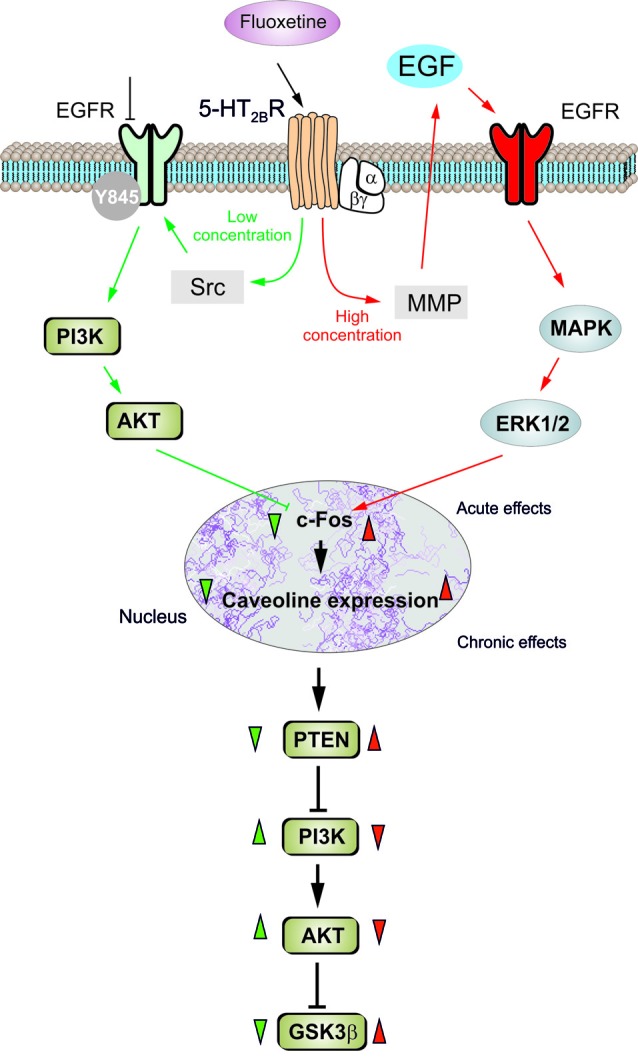
Schematic illustration of biphasic concentration-dependent regulation of Cav-1 gene expression and GSK-3β activity by fluoxetine in astrocytes. Acute treatment with fluoxetine at low concentrations (green arrows) stimulates Src which phosphorylates EGFRs and activates PI3K/AKT signal pathway. The AKT phosphorylation by fluoxetine at low concentrations inhibits cFos gene expression, and subsequently decreases Cav-1 gene expression (chronic effects) that in turn, decreases membrane content of PTEN, induces phosphorylation and stimulation of PI3K and elevates glycogen synthase kinase 3β (GSK-3β) phosphorylation thus suppressing its activity. At higher concentrations fluoxetine (red arrows) stimulates metalloprotinase and induces shedding of growth factor which stimulates EGFRs and activates MAPK/ERK_1/2_ signal pathway. The ERK_1/2_ phosphorylation by fluoxetine at high concentrations stimulates cFos gene expression, and subsequently increases Cav-1 gene expression (chronic effects), that acts on PTEN/PI3K/AKT/GSK-3β in an inverse fashion (Summary of results in the present work and in Bai et al., 2017).

In conclusion, fluoxetine at low concentrations stimulates Src which, phosphorylates EGFRs and activates PI3K/AKT signal pathway, but at high concentrations stimulates metalloproteinase and induces shedding of growth factor which stimulates EGFRs and activates MAPK/ERK_1/2_ signaling pathway (Li et al., 2008; Figure 7). Mechanisms underlying biphasic concentration-dependent regulation of Cav-1 gene expression by fluoxwtine in astrocytes are: (i) inhibition of cFos gene expression by AKT at lower concentrations; and (ii) increase of c-Fos gene expression by ERK_1/2_ at higher concentrations. These opposing effects of PI3K/AKT and MAPK/ERK_1/2_ signaling pathways are fundamental for the biphasic concentration-dependent regulation of GSK3β activity by fluoxetine (Bai et al., 2017). Since the complex roles of Cav-1, the effects of fluoxetine on regulation of Cav-1 gene expression may significantly affect astrocytic functions and signals. Bi-phasic regulation of Cav-1 gene expression in astrocytes, as well as other types of cells in peripheral tissues may contribute to both therapeutic and side effects of the drug. Selective inhibition of PI3K/AKT or MAPK/ERK_1/2_ signal pathway may provide opportunity to avoid non-therapeutic effects of the drug.

## Author Contributions

LP conceived and designed the experiments. BL, SJ, TY, LY and CH performed the experiments. BL, SJ, LY and LP analyzed the data. AV, LP and BL drafted the manuscript, which was critically revised and finally approved by LP and AV. All authors agree to be accountable for all aspects of the work in ensuring that questions related to the accuracy or integrity of any part of the work are appropriately investigated and resolved.

## Conflict of Interest Statement

The authors declare that the research was conducted in the absence of any commercial or financial relationships that could be construed as a potential conflict of interest.

## Supplementary Material

FIGURE S1 Representative original images for the antibodies used in this study. **(A)** p-ERK, **(B)** ERK, **(C)** p-AKT, **(D)** AKT, **(E)** c-Fos and FosB and **(F)** Cav-1.
