# Supplementary figures and images for: Biphasic Regulation of Caveolin-1 Gene Expression by Fluoxetine in Astrocytes: Opposite Effects of PI3K/AKT and MAPK/ERK Signaling Pathways on c-fos
Source: Front Cell Neurosci. 2017 Oct 31;11:335. doi: 10.3389/fncel.2017.00335 (PMC5671492; doi:10.3389/fncel.2017.00335)

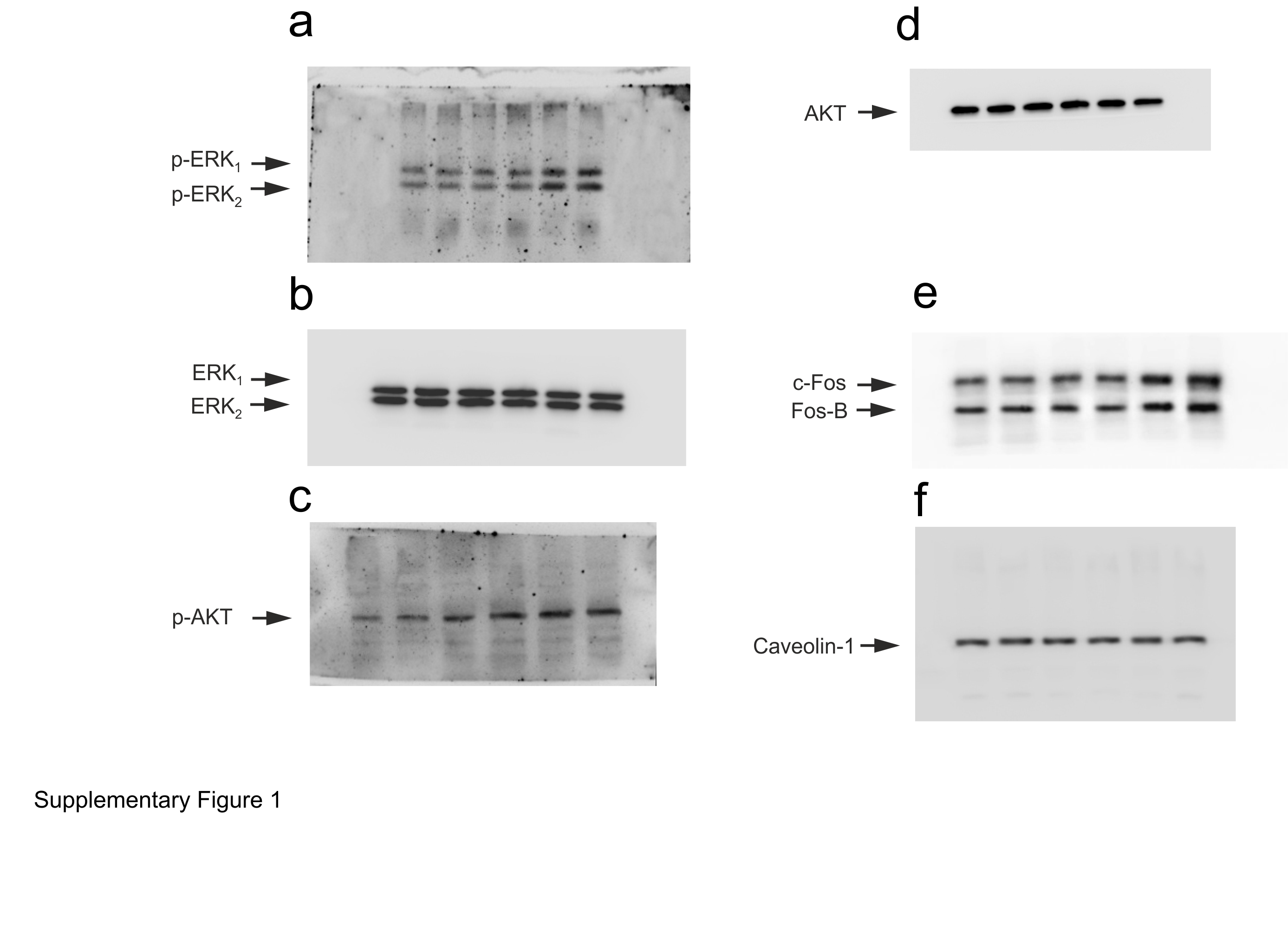

Supplement: FIGURE S1 — Representative original images for the antibodies used in this study. (A) p-ERK, (B) ERK, (C) p-AKT, (D) AKT, (E) c-Fos and FosB and (F) Cav-1. [file Image_1.tif]
